# Supplementary material for: A new small-bodied ornithopod (Dinosauria, Ornithischia) from a deep, high-energy Early Cretaceous river of the Australian–Antarctic rift system
Source: PeerJ. 2018 Jan 11;5:e4113. doi: 10.7717/peerj.4113 (PMC5767335; doi:10.7717/peerj.4113)
Supplement: Supplemental Information 1 [file peerj-06-4113-s001.pdf]

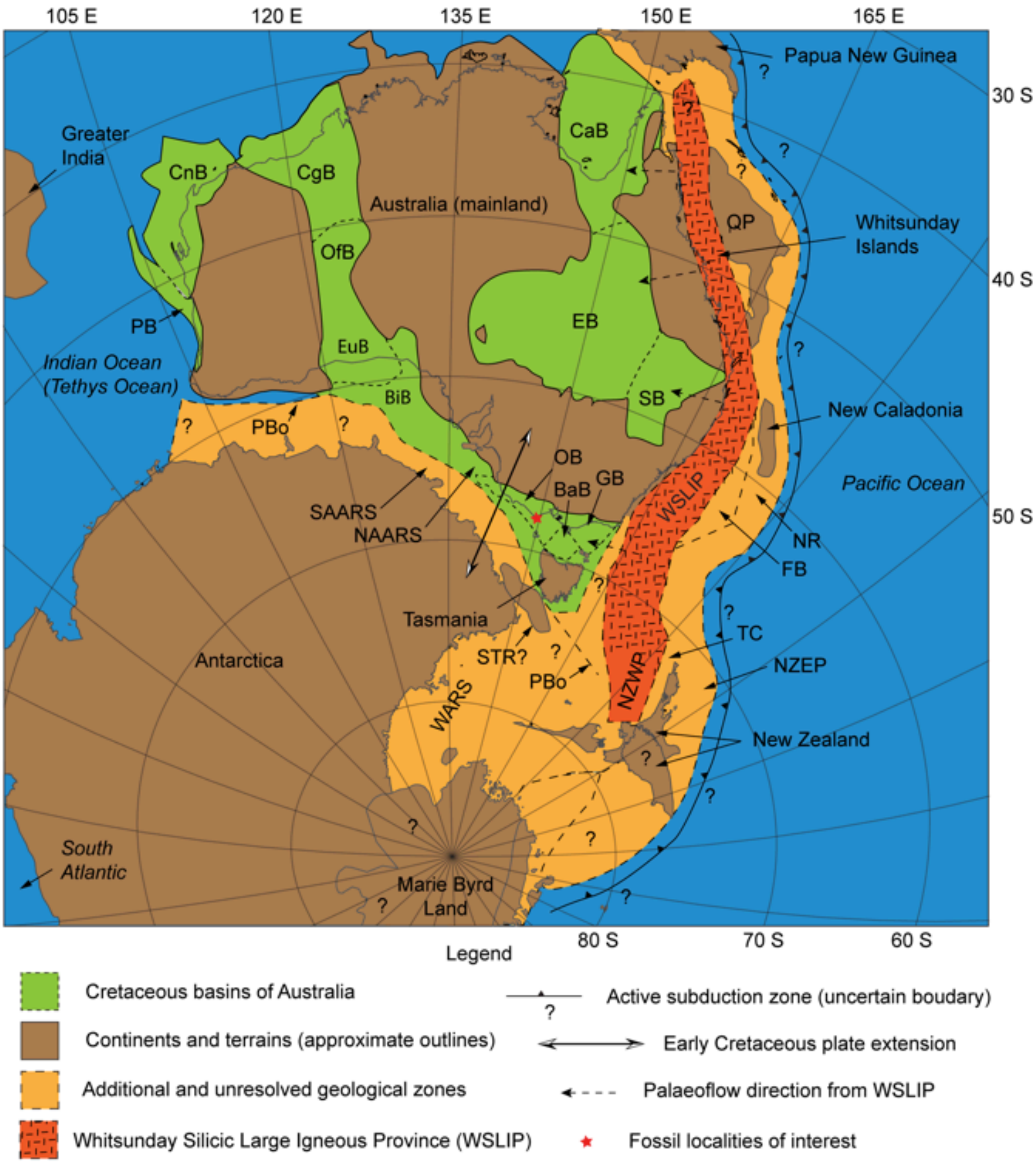

Figure S1. Map of East Gondwana at ~113 Ma in the region of Australia and Antarctica. Reconstruction using GPlates (Müller, Gurnis & Torsvik, 2012; Seton et al., 2012) and geological features based on Willcox & Stagg (1990); Dettmann et al. (1992); Bryan et al. (1997); Gaina et al. (1998); Bryan et al. (2000); Hill & Moore (2001); Norvick & Smith (2001); Bradshaw et al. (2003);

Wandres & Bradshaw (2005); Bryan (2007); Norvick et al. (2008); Matthews et al. (2015); Mortimer et al. (2017). Abbreviations: BaB, Bass Basin; BiB, Bight Basin; CaB, Carpentaria Basin; CgB, Canning Basin; CnB, Carnarvon Basin; EB, Eromanga Basin; EuB, Eucla Basin; FB, Fairway Basin; GB, Gippsland Basin; NAARS, northern region of the Australian-Antarctic rift system; NR, Norfolk Ridge; NZEP, New Zealand Eastern Province; NZWP, New Zealand Western Province; OfB, Officer Basin; OB, Otway Basin; PB, Perth Basin; PBo, Extensional plate boundary; QP, Queensland Plateau; SAARS, southern region of the Australian-Antarctic rift system; SB, Surat Basin; STR, South Tasman Rise; TC, Tutoko Complex; WARS, Western Antarctic Rift System; and WSLIP, Whitsunday Silicic Large Igneous Province.

References

Bradshaw BE, Rollet N, Totterdell JM, Borissova I. 2003. A revised structural framework for frontier basins on the southern and southwestern Australian continental margin. Canberra: Geoscience Australia. p 44.

Bryan SE. 2007. Silicic large igneous provinces. *Episodes* 30:20–31.

Bryan SE, Constantine AE, Stephens CJ, Ewart A, Schon RW, Parianos J. 1997. Early Cretaceous volcano-sedimentary successions along the eastern Australian continental margin: Implications for the break-up of eastern Gondwana. *Earth and Planetary Science Letters* 153:85–102.

Bryan SE, Ewart A, Stephens CJ, Parianos J, Downes PJ. 2000. The Whitsunday Volcanic Province, Central Queensland, Australia: lithological and stratigraphic investigations of a silicic-dominated large igneous province. *Journal of Volcanology and Geothermal Research* 99:55–78.

Dettmann ME, Molnar RE, Douglas JG, Burger D, Fielding C, Clifford HT, Francis J, Jell P, Rich T, Wade M, Vickers-Rich P, Pledge N, Kemp A, Rozefeld A. 1992. Australian Cretaceous terrestrial faunas and floras: biostratigraphic and biogeographic implications. *Cretaceous Research* 13:207–262.

Gaina C, Müller DR, Royer J-Y, Stock J, Hardebeck J, Symonds P. 1998. The tectonic history of the Tasman Sea: A puzzle with 13 pieces. *Journal of Geophysical Research: Solid Earth* 103:12413–12433.

Hill PJ, Moore AMG. 2001. Geological framework of the South Tasman Rise and East Tasman Plateau. Canberra: Geoscience Australia. p 35.

Matthews KJ, Williams SE, Whittaker JM, Müller RD, Seton M, Clarke GL. 2015. Geologic and kinematic constraints on Late Cretaceous to mid Eocene plate boundaries in the southwest Pacific. *Earth-Science Reviews* 140:72–107.

Mortimer N, Campbell HJ, Tulloch AJ, King PR, Stagpoole VM, Wood RA, Rattenbury MS, Sutherland R, Adams CJ, Collot J, Seton M. 2017. Zealandia: earth's hidden continent. *GSA Today* 27:27–25.

Müller RD, Gurnis M, Torsvik T. 2012. GPlates 1.2.0. *Available at* http://www.gplates.org/index.html.

Norvick MS, Langford RP, Rollet N, Hashimoto T, Higgins KL, Morse MP. 2008. New insights into the evolution of the Lord Howe Rise (Capel and Faust basins), offshore eastern Australia, from terrane and geophysical data analysis. In: Blevin JE, Bradshaw BE, Uruski C, eds. *Eastern Australasian Basins Symposium III: Energy security for the 21st century*: Petroleum Exploration Society of Australia Special Publications, 291–310.

Norvick MS, Smith MA. 2001. Mapping the plate tectonic reconstructions of southern and southeastern Australia and implications for petroleum systems. *APPEA journal* 41:15–35.

Seton M, Müller RD, Zahirovic S, Gaina C, Torsvik T, Shephard G, Talsma A, Gurnis M, Turner M, Maus S, Chandler M. 2012. Global continental and ocean basin reconstructions since 200 Ma. *Earth-Science Reviews* 113:212–270.

Wandres AM, Bradshaw JD. 2005. SW Pacific margin of Gondwana from conglomeratic rocks for the configuration of the New Zealand tectonostratigraphy and implications. *Geological Society, London, Special Publications* 246:179–216.

Willcox JB, Stagg HMJ. 1990. Australia's southern margin: a product of oblique extension. *tectonophysics* 173:269–281.
