# Supplementary material for: A new small-bodied ornithopod (Dinosauria, Ornithischia) from a deep, high-energy Early Cretaceous river of the Australian–Antarctic rift system
Source: PeerJ. 2018 Jan 11;5:e4113. doi: 10.7717/peerj.4113 (PMC5767335; doi:10.7717/peerj.4113)
Supplement: Supplemental Information 2 [file peerj-06-4113-s002.pdf]

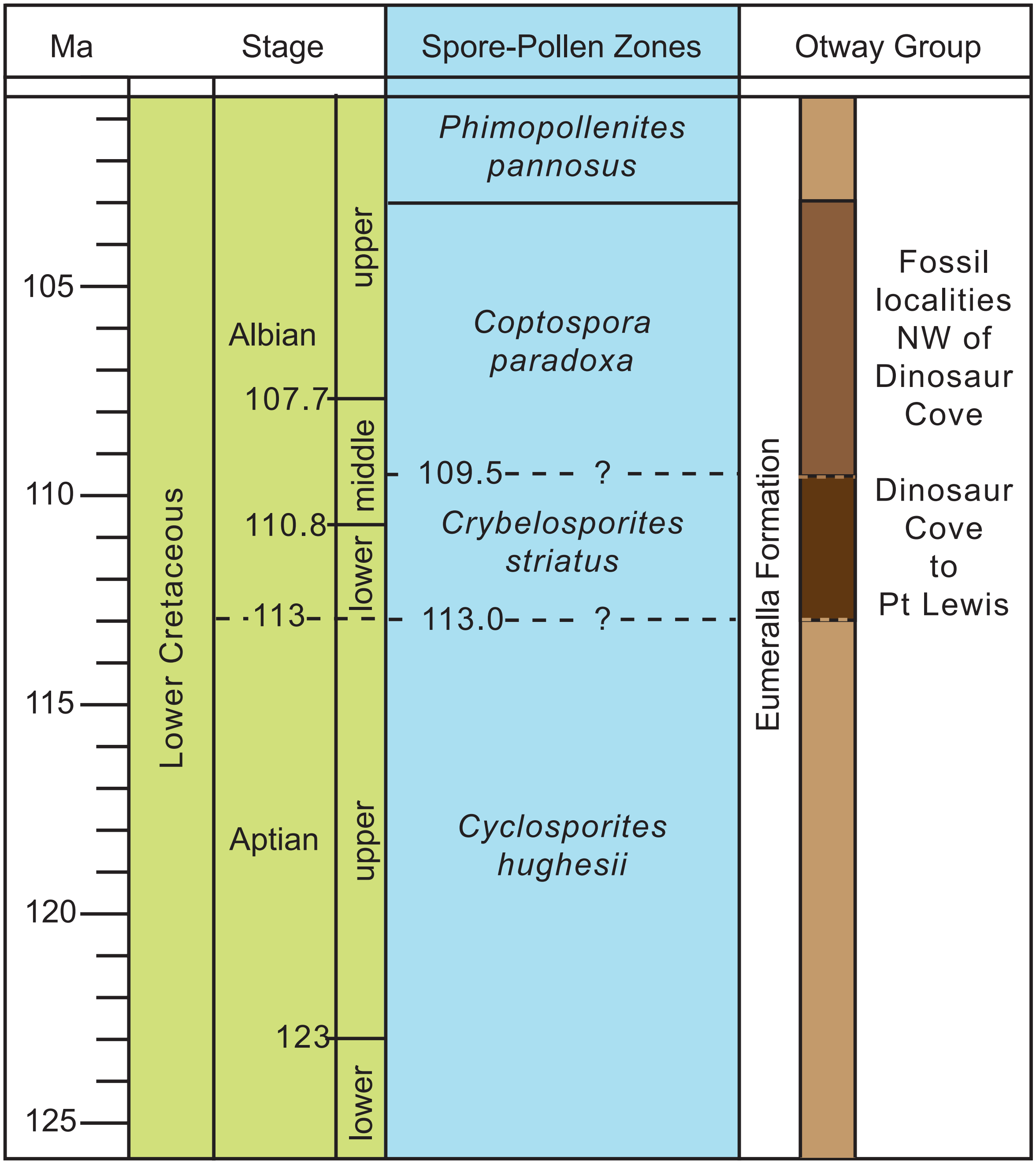


**Figure S2**. Relative ages of Eumeralla Formation fossil vertebrate localities. Spore-pollen zones following Helby, Morgan & Partridge (1987) with revised age estimates following Gradstein, Ogg & Schmitz (2012), Wagstaff, Gallagher & Trainor (2012) and Korasidis et al. (2016).

# References

Gradstein FM, Ogg G, Schmitz M. 2012. *The Geologic Time Scale 2012, 2-Volume Set*: Elsevier.

Helby R, Morgan R, Partridge AD. 1987. A palynological zonation of the Australian Mesozoic. *Memoirs of the Association of Australasian Palaeontologists* 4:1–94.

Korasidis VA, Wagstaff BE, Gallagher SJ, Duddy IR, Tosolini AMP, Cantrill DJ, Norvick MS. 2016. Early angiosperm diversification in the Albian of southeast Australia: implications for flowering plant radiation across eastern Gondwana. *Review of Palaeobotany and Palynology* 232:61–80.

Wagstaff BE, Gallagher SJ, Trainor JK. 2012. A new subdivision of the Albian spore-pollen zonation of Australia. *Review of Palaeobotany and Palynology* 171:57–72.
