# Supplementary figures and images for: A new small-bodied ornithopod (Dinosauria, Ornithischia) from a deep, high-energy Early Cretaceous river of the Australian–Antarctic rift system
Source: PeerJ. 2018 Jan 11;5:e4113. doi: 10.7717/peerj.4113 (PMC5767335; doi:10.7717/peerj.4113)

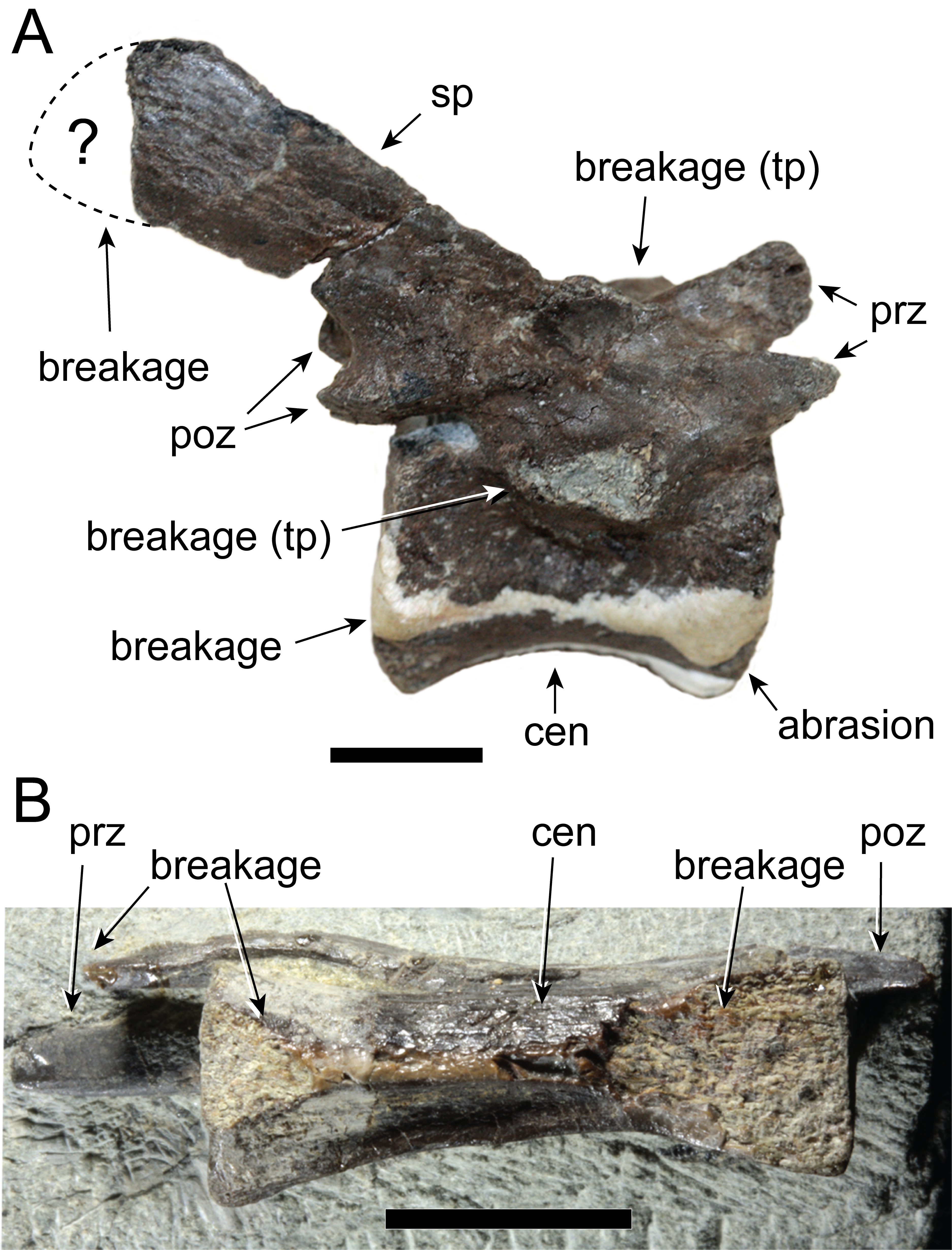

Supplement: Supplemental Information 3 — (A) NMV P228342 in right dorsolateral view. (B) NMV P229456 in left lateroventral view. Abbreviations: cen, centrum; poz, postzygapophysis; prz, prezygapophysis; sp, spinal process; tp, transverse process. Scale bar 1 cm. [file peerj-06-4113-s003.png]

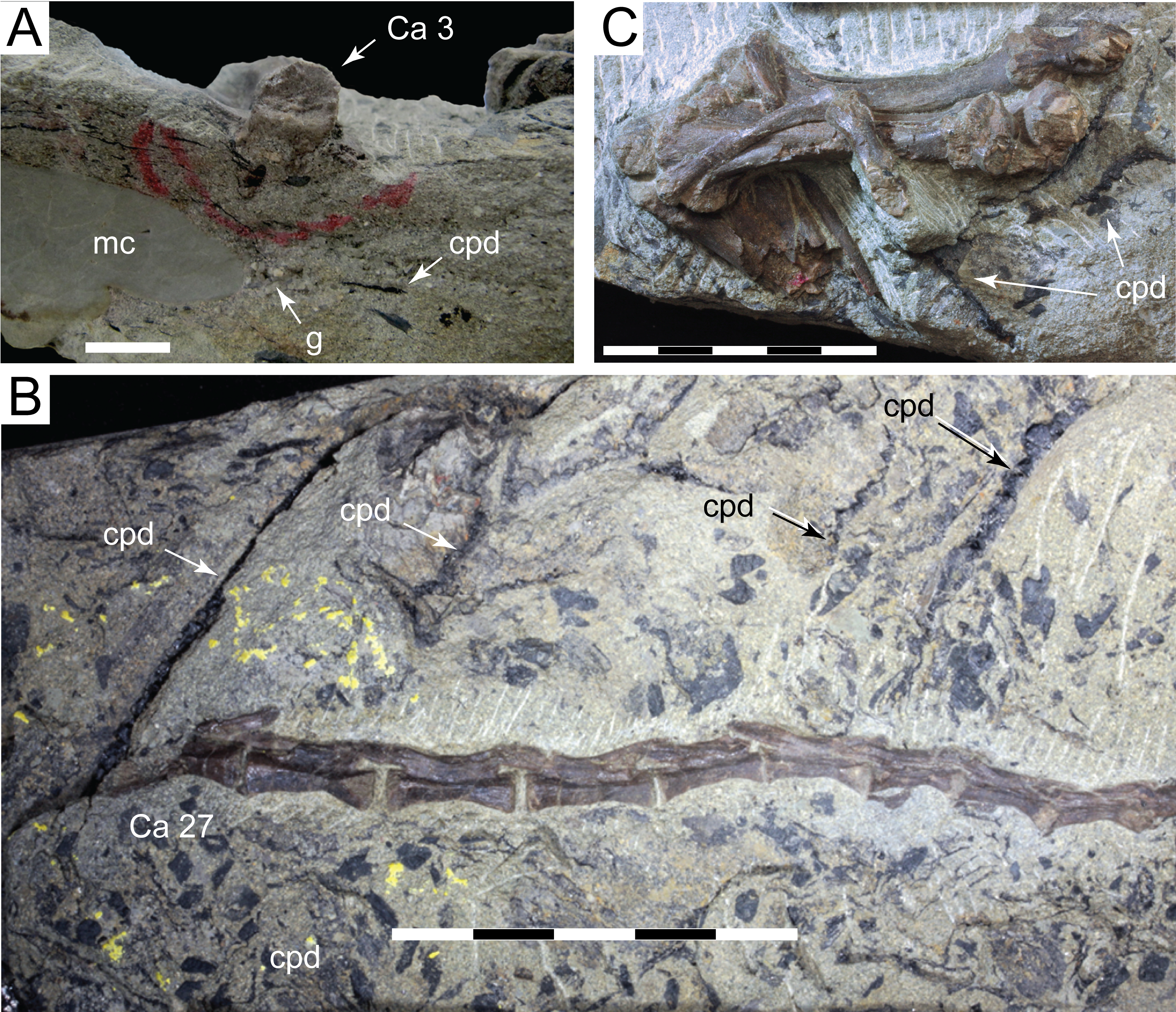

Supplement: Supplemental Information 4 — (A) Eroded vertical surface on block ‘B1’ looking north in the region of the anterior caudal vertebrae. (B) Top view of block ‘B1’ in the region of the right pes. (C) Top view of block ‘B5’ in the region of the posterior caudal vertebrae. Abbreviations: Ca #, designated caudal vertebra and position; cpd, coalified plant debris; g, gravel/grit; mc, mudrock clast. Scale bar in A, 1 cm. Scale increments in B–C, 1 cm. [file peerj-06-4113-s004.png]

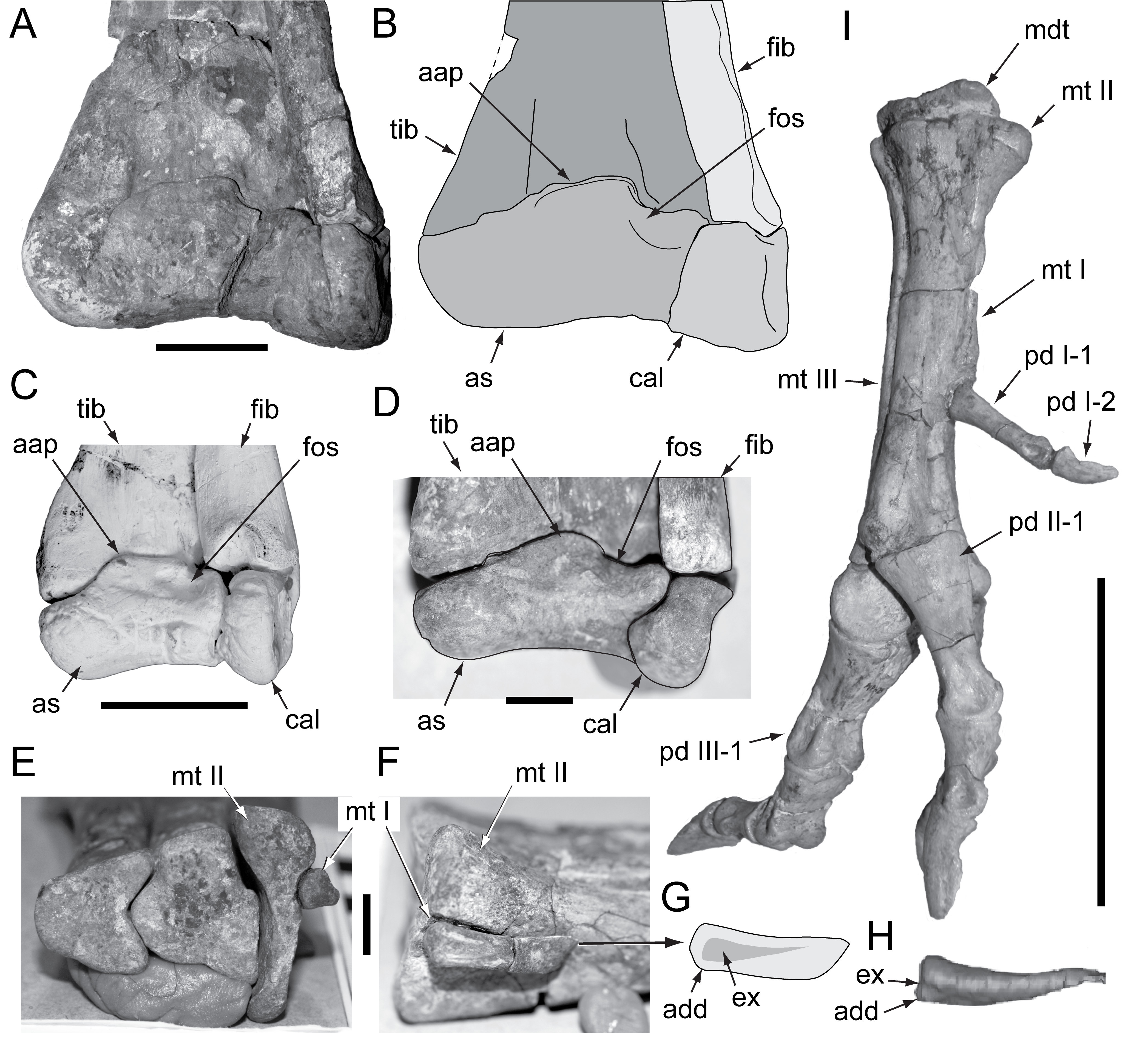

Supplement: Supplemental Information 5 — A–B, distal left crus and proximal tarsus of the Muttaburrasaurus langdoni holotype (QM F6140) in anterior view: (A) image; and (B) schematic. (C) Distal left crus and proximal tarsus of D. altus (YPM 1876, cast) in anterior view. D–G, Anabisetia (MCF-PVPH-74): (D) distal left crus and proximal tarsus in anterior view; (E) left metatarsus in proximal view; (F) proximal region of left metatarsus in medial view; and (G) schematic of postulated distal mt I in plantomedial view (shown separated from the proximal end of mt II). (H) CT model of distal left mt I of the D. pickeringi holotype (NMV P221080) in in plantomedial view. (I) Right pes of Anabisetia (MCF-PVPH-75) in medial view. Abbreviations: aad, adductor surface; aap, anterior ascending process of astragalus; as, astragalus; cal, calcaneum; ex, extensor groove; fib, fibula; fos, fossa; mt #, metatarsal and position; pd #, pedal phalanx, number and phalanx position; tib, tibia. Scale bars: A and G, 10 cm; B, 5 cm; C–F, 1 cm. [file peerj-06-4113-s005.png]
