# Supplementary material for: A new small-bodied ornithopod (Dinosauria, Ornithischia) from a deep, high-energy Early Cretaceous river of the Australian–Antarctic rift system
Source: PeerJ. 2018 Jan 11;5:e4113. doi: 10.7717/peerj.4113 (PMC5767335; doi:10.7717/peerj.4113)
Supplement: Supplemental Information 7 [file peerj-06-4113-s007.pdf]

Figure S7

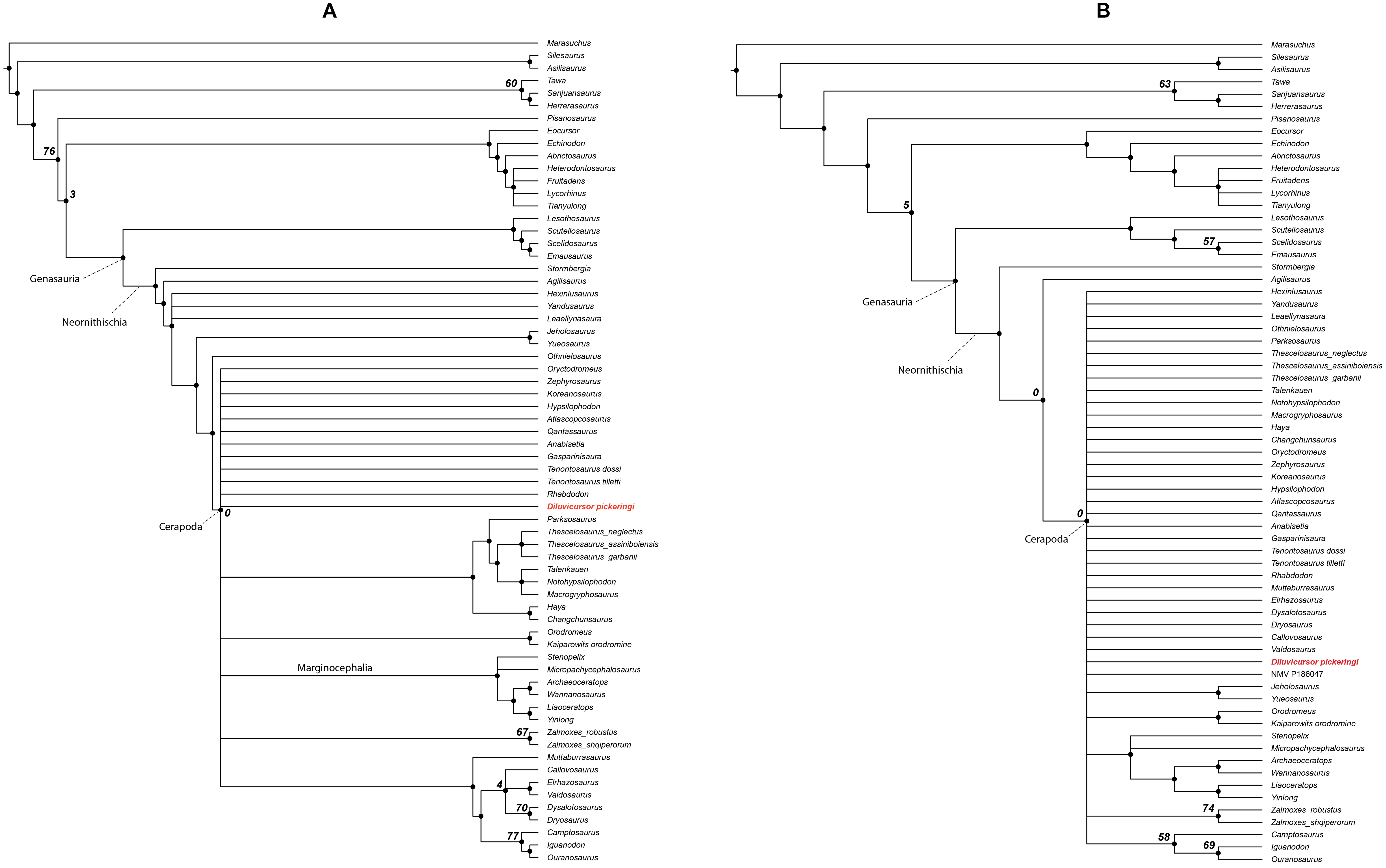

Figure S7. *Diluvicursor pickeringi* in strict consensus trees derived from the matrix published by Boyd (2015), showing: **(A)** strict consensus of all OTUs from Boyd (2015) + *Diluvicursor pickeringi*, without modification of character scores; and **(B)** strict consensus of all OTUs from Boyd (2015) + *Diluvicursor pickeringi* + modifications to the scores of four OTUs (see below) + NMV P186047. Numbers reported at the selected nodes of interest are bootstrap resampling percentages.

In regards to the original Traditional Search parameters of Boyd (2015): 10,000 replications at 10,000 tree-holds per replicate require a TNT tree-holding capacity of 100,000,000, and an unrealistic time for the search analysis to be completed. Herein, both searches (**A**, **B**) were re-run with 5,000 replications (4 trees held per replicate) under the TBR branch-swapping algorithm. As per Boyd (2015), all characters were unordered. Bootstrap support was calculated for the consensus trees using 100 pseudoreplications, using ‘Frequency Distributions’ in TNT (this takes into account resampling that also contradicts recovered clades in the consensus [Goloboff et al. 2003]).

The analysis yielding the consensus that is shown in **A** is derived from 48 most parsimonious trees with a length of 872 steps. The consensus shows that Boyd’s 2015 dataset could only resolve *Diluvicursor pickeringi* within Cerapoda as part of a broad polytomy with most other well-established cerapodan OTUs; this composition of the cerapodan node was unsupported by Bootstrap resampling.

In the secondary search of the Boyd (2015) matrix, the following modifications to character scores were made: *Atlascopcosaurus loadsi*, maxillary scores were maintained (i.e., Boyd, 2015, characters 75, 79–80, 117, 119–122, 125, 127–129, 131, 134); however, all dentary scores were excluded (characters 118, 124, 133, 135–136, 138–139: -->?), as conclusively associated dental material is lacking for *Atlascopcosaurus loadsi* (Herne, 2013). A maxillary tooth character scored for *Qantassaurus intrepidus*, which is known only from the dentary, was altered (Boyd, 2015, character 132: 4-->?) as no maxillary material is associated with this taxon. The postcranial material originally included by Boyd (2015) within *Leaellynasaura amicagraphica* was removed from this OTU. Based on the original scoring, it is the indeterminate Victorian postcranium NMV P186047 (sensu Herne, 2013; Herne, Tait & Salisbury, 2016), and it was added to the matrix as an independent OTU. This second analysis yielded a consensus (shown in **B**) from 484 most parsimonious trees with a length of 869 steps. *Diluvicursor pickeringi* was recovered within a weakly supported Cerapoda as part of a broad polytomy with most other OTUs. Notably, the corrections to the scores of the aforementioned important Victorian OTUs resulted in the consensus tree exhibiting almost no resolution, with only *Scelidosaurus*+*Emausaurus*, Ankylopollexia and *Zalmoxes* spp. recovered as monophyletic groups with significant support.

Figure S7

In the strict consensus of the first analysis (**A**), node Cerapoda is supported by 10 optimized synapomorphies from the character list of Boyd (2015): 71(1), 77(1), 106(1), 123(1), 135(1), 139(1), 169(1), 193(2), 200(1), and 213(1). Notably, this list of characters differs substantially from the original analysis prior to the inclusion of *D. pickeringi* (Boyd, 2015, supp. table 4 showing characters diagnosing 'Node 12 → Node 13' [=Cerapoda]: 26(1), 52(1), 112(2), 120(1), 123(1), 124(1), 252(0). Within the amended analysis, the inclusion of *D. pickeringi* was unstable. With the inclusion of *D. pickeringi*, the membership of Cerapoda differed from that of the original analysis of Boyd (2015), had relatively reduced internal resolution, and was described by a different suite of characters.

This problem is exacerbated in the second analysis, with corrections to the Eumeralla Formation taxa further resulting in a much less resolved internal consensus for node Cerapoda (**B**). Here, 'Cerapoda' is described by 21 'synapomorphies', presumably due to the presence of fewer internal nodes where some of these characters may have otherwise informed: 57(1), 72(1), 81(1), 85(1), 114(1), 120(1), 121(1), 123(1), 139(1), 148(2), 183(1), 184(0), 189(1), 191(1), 202(0), 212(1), 217(2), 218(1), 222(1), 232(1), and 250(1).

References:

Boyd CA. 2015. The systematic relationships and biogeographic history of ornithischian dinosaurs. *PeerJ* 3:e1523, doi:10.7717/peerj.1523

Goloboff PA, Farris JS, Källersjö M, Oxelman B, Ramirez MI, Szumik CA. 2003. Improvements to resampling measures of group support. *Cladistics* 19:324–332.

Herne MC. 2014. Anatomy, Systematics and Phylogenetic Relationships of the Early Cretaceous Ornithopod Dinosaurs of the Australian-Antarctic Rift System, PhD dissertation. The University of Queensland.

Herne MC, Tait AM, Salisbury SW. 2016. Sedimentological reappraisal of the *Leaellynasaura amicagraphica* (Dinosauria, Ornithopoda) holotype locality in the Lower Cretaceous of Victoria, Australia, with taphonomic implications for the taxon. *New Mexico Museum of Natural History and Science Bulletin* 71:121–148.
