# Supplementary material for: A new small-bodied ornithopod (Dinosauria, Ornithischia) from a deep, high-energy Early Cretaceous river of the Australian–Antarctic rift system
Source: PeerJ. 2018 Jan 11;5:e4113. doi: 10.7717/peerj.4113 (PMC5767335; doi:10.7717/peerj.4113)
Supplement: Supplemental Information 8 [file peerj-06-4113-s008.pdf]

Figure S8

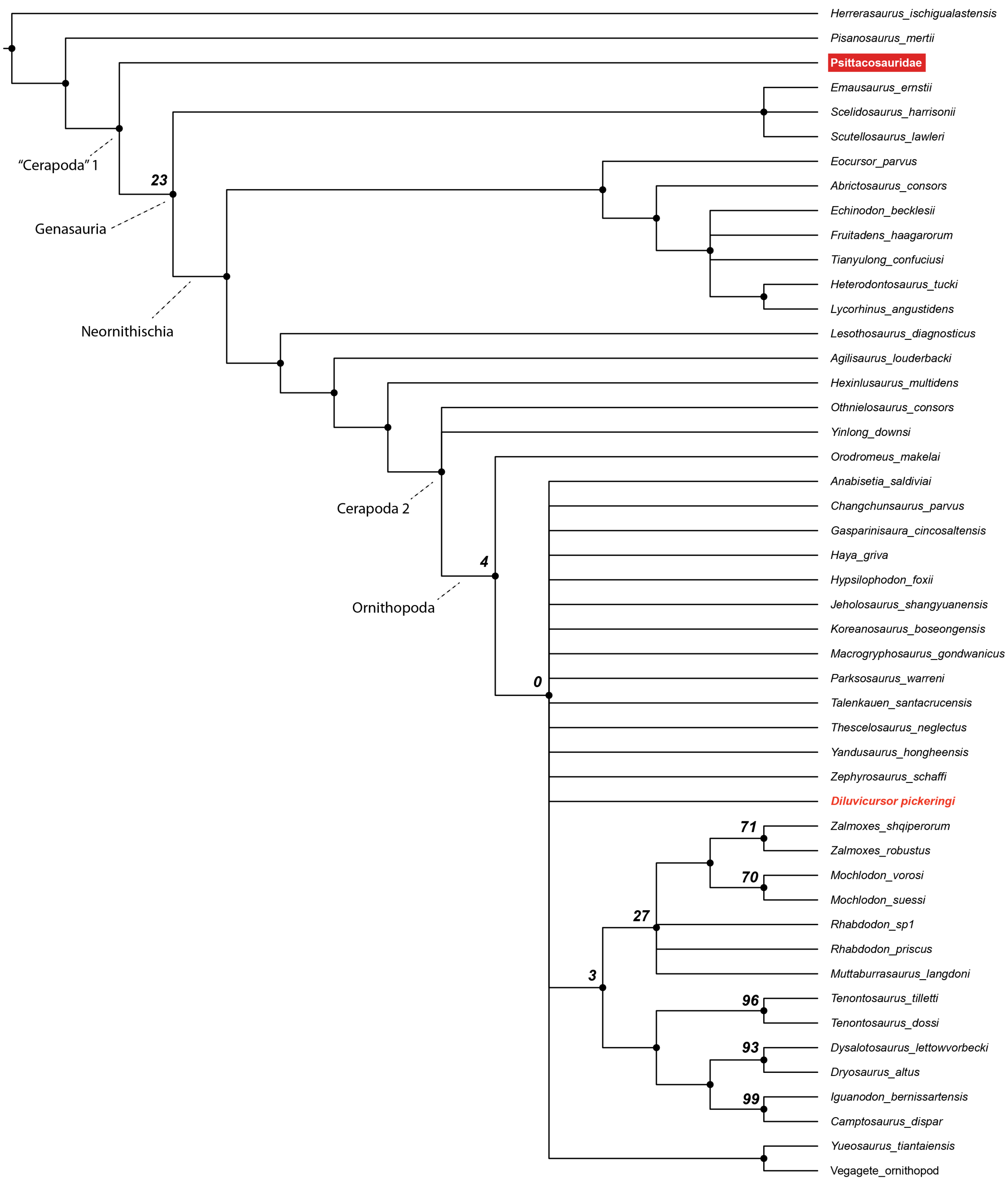

Figure S8. *Diluvicursor pickeringi* in the strict consensus tree derived from the matrix published by Dieudonné et al. (2016). Numbers reported at the selected nodes of interest are bootstrap resampling percentages.

In accordance with the original Traditional Search parameters of Dieudonné et al. (2016), the search was re-run with 1,000 replications (10 trees held per replicate) under the TBR branch-swapping algorithm. As per Dieudonné et al. (2016), all characters were unordered except for 9 characters: "#126, #159, #167, #168, #170, #222, #223, #225, and #262". Bootstrap support was calculated for the consensus trees using 1000 pseudoreplications, using 'Frequency Distributions' in TNT (this takes into account resampling that also contradicts recovered clades in the consensus

Figure S8

[Goloboff et al. 2003]). The consensus was derived from 90 most parsimonious trees with lengths of 857 steps. *D. pickeringi* was recovered as an ornithopodan more nested than *Orodromeus makelai*, as part of a large polytomy. 7 character state changes were recorded for the node labelled Ornithopoda, while 16 were indicated for the node of comprising *D. pickeringi* and other OTUs more nested than *Orodromeus*. However, bootstrap support for both groups was insignificant, and *D. pickeringi* itself was scored for only 1 of these 23 potential synapomorphies: character 283: 1 --> 0.

A re-analysis of the dataset, where all characters were set to unordered, resulted in a consensus where Psittacosauridae was found in a large basal polytomy with other OTUs (including *Yinlong downsi*) at the node labelled 'Cerapoda 2' (with Ornithopoda not being recovered) (not reproduced here). In the dataset published by Dieudonné et al. (2016) marginocephalians are represented by only two OTUs, potentially limiting the scope for a non-ornithopodan placement for many putative basal cerapods. This emphasizes that the ornithopodan membership of *Diluvicursor pickeringi* (and other suggested ornithopods) in the strict consensus with ordered characters is entirely uncertain without an increased representation of marginocephalian OTUs included.

With *Diluvicursor pickeringi* included in the Dieudonné et al (2016) dataset (i.e., with 9 additive characters), Ornithopoda according to the strict consensus was supported by 5 optimized synapomorphies. None of these, however, could be actually be scored in *D. pickeringi*, due to its incompleteness. These synapomorphies are: 34(1), 170(2), 179(1), 226(1), and 249(1). The node *Orodromeus* + MDT, which is largely unresolved internally, was diagnosed by up to 16 characters, emphasizing a similar issue as reported in Supplemental Figure 7.

References:

Dieudonné P-E, Tortosa T, Torcida Fernández-Baldor F, Canudo JI, Díaz-Martínez I. 2016. An unexpected early rhabdodontid from Europe (Lower Cretaceous of Salas de los Infantes, Burgos Province, Spain) and a re-examination of basal iguanodontian relationships. PLoS ONE. 11(6):e0156251, doi:10.1371/journal.pone.0156251.

Goloboff PA, Farris JS, Källersjö M, Oxelman B, Ramirez MI, Szumik CA. 2003. Improvements to resampling measures of group support. *Cladistics* 19:324–332.
