# Supplementary material for: A new small-bodied ornithopod (Dinosauria, Ornithischia) from a deep, high-energy Early Cretaceous river of the Australian–Antarctic rift system
Source: PeerJ. 2018 Jan 11;5:e4113. doi: 10.7717/peerj.4113 (PMC5767335; doi:10.7717/peerj.4113)
Supplement: Supplemental Information 9 [file peerj-06-4113-s009.pdf]

Figure S9

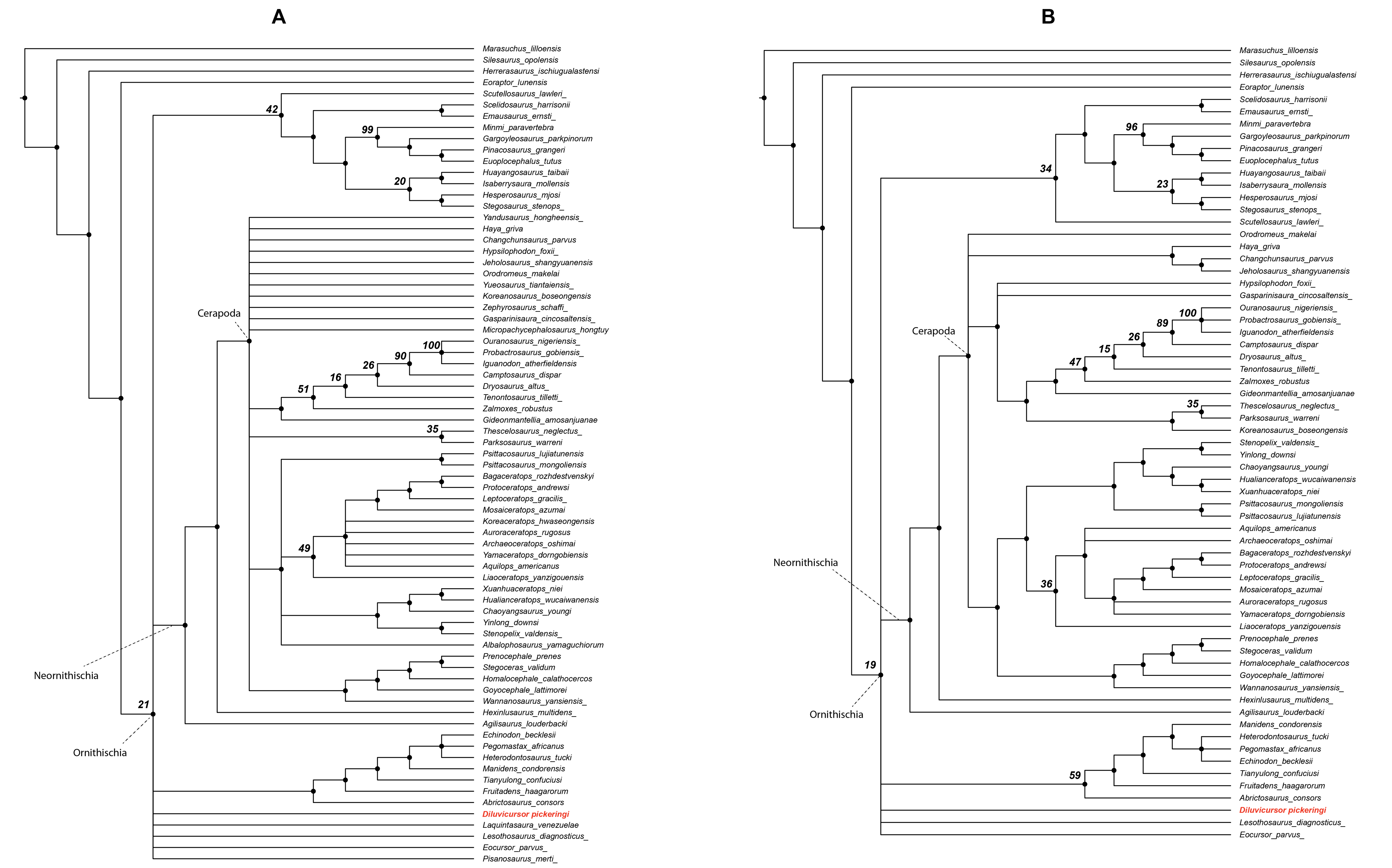

Figure S9. *Diluvicursor pickeringi* in strict consensus trees derived from the matrix published by Han et al. (2017), showing: **(A)** strict consensus of all OTUs in Han et al. (2017) + *Diluvicursor pickeringi*; and **(B)** strict consensus of all OTUs in Han et al. (2017) + *Diluvicursor pickeringi*, after eight OTUs were pruned *a posteriori* (see Han et al. 2017, caption for fig. 16). Numbers reported at selected nodes are bootstrap resampling percentages.

In accordance with the original Traditional Search parameters of Han et al. (2017), the search (yielding both **A**+**B**) was re-run with 1,000 replications (100 trees held per replicate) under the TBR branch-swapping algorithm. As per Han et al. (2017) all characters were unordered except for 21 characters: "2, 23, 31, 39, 125, 163, 196, 203, 204, 222, 227, 238, 243, 247, 268, 292, 296, 302, 306, 320, 361". Bootstrap support was calculated for both consensus trees using 100 pseudoreplications (as per Han et al., 2017). The analysis yielding the consensus that is shown in **A** is derived from 30100 most parsimonious trees with lengths of 1215 steps. This un-pruned consensus shows that the Han et al. (2017) dataset could resolve *Diluvicursor pickeringi* only within Ornithischia as part of a polytomy with basal ornithischians, Heterodontosauridae, Thyreophora and neornithischians, with the ornithischian node being weakly supported by bootstrap resampling. The pruned consensus tree (**B**) also shows resolution of *Diluvicursor pickeringi* within Ornithischia as part of a polytomy, with the ornithischian node again being weakly supported by bootstrap resampling.

Reference:

Goloboff PA, Farris JS, Källersjö M, Oxelman B, Ramirez MI, Szumik CA. 2003. Improvements to resampling measures of group support. *Cladistics* 19:324–332.

Han F, Forster CA, Xu X, Clark JM. In Press, 2017. Postcranial anatomy of *Yinlong downsi* (Dinosauria: Ceratopsia) from the Upper Jurassic Shishugou Formation of China and the phylogeny of basal ornithischians. *Journal of Systematic Palaeontology* 29 pp., doi.org/10.1080/14772019.2017.1369185
