# Supplementary material for: A new small-bodied ornithopod (Dinosauria, Ornithischia) from a deep, high-energy Early Cretaceous river of the Australian–Antarctic rift system
Source: PeerJ. 2018 Jan 11;5:e4113. doi: 10.7717/peerj.4113 (PMC5767335; doi:10.7717/peerj.4113)
Supplement: Supplemental Information 10 — Abbreviations: ETRW, Eric the Red West. [file peerj-06-4113-s010.pdf]

Table S1. Fossil taxa/materials examined, compared or mentioned in this work, with information on occurrence, primary literature sources and additional image resources utilized.

**Taxon/specimen**	**Literature sources**	**Occurrence/provinance**	**Materials examined directly(*), other resources utilized and comments.**
Agilisaurus louderbacki	Peng (1992)	Bathonian-Callovian, Sichuan Province, People's Republic of China (Norman et al., 2004).	Figure S6/Table S3 (ZDM 6011; following Peng, 1992, table 4).
Anabisetia saldiviai	Coria & Calvo (2002), Cambiaso (2007).	Cenomanian, Neuquén, Patagonia, Argentina.	*MCF-PVPH-74, partial cranium and postcranium; *MCF-PVPH-75, partial postcranium. Figure S6/Table S3 (MCF-PVPH-75; following Cambiaso, 2007, appendix II, plate 5)
Camptosaurus dispar	Gilmore (1909).	Kimmeridgian–Tithonian, western United States and Oklahoma, South Central United States (Norman, 2004).	Figures 28, S6/Tables S2–S3 (USNM 4277, 4282; following Gilmore, 1909, figs 18, 35 table p. 266).
***Cumnoria prestwichii* (also known as *Camptosaurus prestwichii*)**	Galton & Powell (1980), McDonald (2011).	Lower Kimmeridgian, Kimmeridge Clay, Oxford, England.	Figure 32 (OUMUK J3373; following Galton & Powell, 1980, fig. 11).
Changchunsaurus parvus	Butler et al. (2011).	Aptian–Cenomanian, Quantou Formation,<br>Songliao Basin, JInlin Province, People's Republic of China.	Study images courtesy R. Butler: JLUM L0403-j-Zn2, proximal right metatarsus. Figure S6/Table S3 (JLUM L0403-j-Zn2; following Butler, 2011, figs 7–8).
***Diluvicursor pickeringi* nov. gen. et sp.**	This investigation	Lower Albian, Eumeralla Formation (Otway Group), Victoria, Australia.	*NMV P221080, holotype, partial postcranium; NMV P229456, referred caudal vertebra.
Drinker nisti	Bakker et al. (1990).	Upper Jurassic, Morrison Formation, Wyoming, United States.	

Dryosaurus altus	Galton (1981).	Kimmeridgian–Tithonian, Morrison Formation, western United States (Norman, 2004).	*YPM 1876, crus and proximal tarsus (casts); *YPM 1882, left pes (cast). Study images courtesy M. Lamanna: CM 21786, right pes; YPM 1882, left and right pedes.
Dysalotosaurus lettowvorbecki	Janensch (1955), Galton (1981), Hübner (2011).	Kimmeridgian, Tendagaru Formation, Mtwara, Tanzania, Eastern Africa (Norman, 2004).	*NHMUK R12282, right metatarsal III; NHMUK R12279, right tibia. Study images courtesy D. Schwartz: MB.R.1397, left mt III; MB.R.1398, left mt II; MB.R.1409, right mt IV. Figure 28/Table S2 (MB.R.1587.4 [HMN dy II], following: Janensch, 1955, fig. 26; Hübner, 2011, fig. 5.1).
Eousdryosaurus nanohallucis	Escaso et al. (2014).	Upper Kimmeridgian, Alcobaça Formation, Portugal.	Figures 28, 32/Table S2 (SHN(JJS)-170, following Escaso et al., 2014, figs 2, 6).
Gasparinisaura cincosaltensis	Coria & Salgado (1996), Salgado, Coria & Heredia (1997).	Conaician–Santonian, Rio Colorado Formation, western Rio Negro Province, Patagonia, Argentina.	*MUCPv-208, partial postcranium; *MUCPv-212, articulated caudal vertebrae; *MUCPv-213, partial hind limbs; *MCS-1, almost complete articulated tail; *MCS-2, articulated right pes; *MCS-3, caudal vertebrae, partial hind limbs.
Haya griva	Makovicky et al. (2011).	Upper Cretaceous (Santonian?), Jaukhlant Formation, Khugenetslavkant locality (Dorngobi Province), eastern Gobi, Mongloia (Eberth et al., 2009).	Figure 28/Table S2 (IGM 100/2015; following Makovicky et al., 2011, fig.3C).
Heterodontosaurus tucki	Santa Luca (1980), Sereno (2012), Galton (2014).	Hettangian-Sinemurian, Upper Elliot Formation, Clarens Fromation, South Africa.	Figure S6/Table S3 (SAM-PK-1332; following Santa Luca, 1980, appendix 1).
Hexinlusaurus multidens	He & Cai (1984).	Bathonian–Callovian, Sichuan Province, People's Republic of China.	

Hypsilophodon foxii	Hulke (1882), Galton (1974).	Barremian–early Aptian, the Isle of Wight, England and La Roja region of Spain (Norman et al., 2004).	*NHMUK R193, partial right hind limb; *NHMUK R196, partial postcranium. Figures 28, 32, 35, S6/Tables S2–S3 (NHMUK R196, R5830; following Galton, 1974, figs 28–29, 57H, table 3).
Iguanodon bernissartensis	Norman (1980).	Valanginian–Aptian, multiple formations, England, France, Belgium, Germany, Spain and Mongolia.	
Jeholosaurus shangyuanensis	Han et al. (2012).	Lower Aptian, Yixian Formation, Lujiatun, Liaoning Province, People's Republic of China.	Figures 28, S6/Tables S2–S3 (IVPP V12542, V15939; following Han et al., 2012, fig. 5A, table A5).
Kangnasaurus coetzeei	Cooper (1985), Haughton (1915).	Upper Cretaceous, Bushmanland, South Africa (de Wit, Ward & Spaggiari, 1992).	Study images courtesy K. Poole: SAM-PK-2731, fragmentary hind limb elements.
Lesothosaurus diagnosticus?	Thulborn (1972), Sereno (1991), Butler (2005).	Hettangian–Sinemurian, Upper Elliot Formation, Lesotho Region, South Africa.	
Macrogryphosaurus gondwanicus	Calvo, Porfiri & Novas (2007).	Maastrichtian, Portezuelo Formation (Neuquén Group), Neuquén Province, Argentina.	*MUCPv–321, partial postcranium.
Mantellisaurus atherfieldensis	Norman (1986).	Valanginian–Aptian, multiple formations, England, France, Belgium, Germany and Spain.	*NHMUK R11521, left and right pedes. Figure 28/Table S2 (IRSNB 1551; following Norman, 1986, fig. 39, appendix 2).
Morrosaurus antarcticus	Rozadilla et al. (2016), Cambiaso (2007).	Lower Maastrichtian, Snow Hill Island Formation/Lopez de Bertodano Formation, James Ross Island, Antarctica.	Figure 32 (MACN Pv-19777; following Rozadilla et al., 2016, fig. 5).

Muttaburrasaurus langdoni	Bartholomai & Molnar (1981), Molnar (1996).	Albian, Mackunda Formation, central western Queensland, Australia.	*QM F6140, holotypic partial postcranium.
**NMV P185992/NMV P185993 (Indeterminate Ornithopoda)**	(Rich & Rich, 1989); Rich & Vickers-Rich (1999), Herne (2009), Herne (2014), Herne, Tait & Salisbury (2016).	Lower Albian, Eumeralla Formation (Otway Group), Dinosaur Cove, southern Victoria, Australia.	*Partial postcranium.
**NMV P186047 (indeterminate Ornithopoda)**	Gross, Rich & Vickers-Rich (1993), Herne (2009), Herne (2014), Herne, Tait & Salisbury (2016).	Lower Albian, Eumeralla Formation (Otway Group), Dinosaur Cove, Victoria, Australia.	*Partial postcranium.
**NMV P221081 *(Spinosauroidea)***	Barrett et al. (2011a).	Lower Albian, Eumeralla Formation (Otway Group), ETRW Sandstone, Victoria, Australia.	Isolated cervical vertebra.
**NMV P228342**	This investigation	Lower Albian, Eumeralla Formation (Otway Group), ETRW Sandstone, Victoria, Australia.	*Isolated caudal vertebra.
Notohypsilophodon comodorensis	Martinez (1998), Ibiricu et al. (2014), Cambiaso (2007).	Cenomanian? Bajo Barreal Formation, Chubut Province, Patagonia, Argentina	*UNPSJB PV942, partial postcranium.

Orodromeus makelai	Scheetz (1999), Brown et al. (2013).	Middle Campanian–Early Maastrichtian, Alberta, western Canada and Montana, western United States (Norman et al., 2004).	Figures 28, S6/Tables S2–S3 (MOR 530, 623; following Scheetz, 1999, fig. 31).
Oryctodromeus cubicularis	Varricchio, Martin & Katsura (2007), Brown et al. (2013), Krumenacker (2017).	Albian-Cenomanian Wayan and Blackleaf formations, Idaho and Montana, western United States.	
Othnielosaurus consors	Galton & Jensen (1973).	Upper Jurassic, Morrison Formation, western Unites States (Galton & Jensen, 1973).	*Cast referred pes, ROM 46240 (BYU ESM-163R).
Ouranosaurus nigeriensis	Taquet (1976).	Upper Aptian, Elrhaz Formation, Niger.	
Parksosaurus warreni	Parks (1926), Brown et al. (2013).	Maastrichtian (70.6 to 65.5 Ma) Horseshoe Canyon Formation, Alberta, Canada (Norman et al., 2004).	*ROM 804, almost complete postcranium.
Talenkauen santacrucensis	Novas, Cambiaso & Ambrosio (2004), Novas (2009), Cambiaso (2007).	Maastrichtian, Pari Aike Formation, Santa Cruz Province, Patagonia, Argentina.	Figure 32 (MPM 10001; following Cambiaso, 2007, fig. 40).
Tenontosaurus tilletti	Ostrom (1970), Forster (1990).	Upper Aptian–middle Albian, Cloverly Formation, Montana, Wyoming, Texas, United States (Norman, 2004).	Figures 28, 32/Table S2 (AMNH 3040, YPM 16338; following Forster, 1990, figs 5, 22).
Thescelosaurus assiniboiensis	Brown, Boyd & Russell (2011).	Maastrichtian, Frenchman Formation, western Sasketchewen, Canada (Brown, Boyd & Russell, 2011).	Figures 32, S6/Table 3 (RSM P1225.1; following Brown et al., 2011, fig. 22).

Thescelosaurus neglectus	Gilmore (1915).	Maastrichtian, Lance Formation, Montana, western United States and western Canada (Norman et al., 2004).	Figure 28/Table S2 (USNM 7757; following Gilmore, 1915, fig. 6).
***Thescelosaurus* sp. (CMN 8537)**	Sternberg (1940)	See for *T. neglectus*	
Valdosaurus canaliculatus	Galton & Taquet (1982), Barrett et al. (2011b), Barrett (2016).	Lower Cretaceous (Berriasian-Barremian), multiple formations, England and Romania	Figure 28/Table S2 (IWCMS 2013.175; following Barrett, 2016, fig. 3C).
Zalmoxes robustus	Weishampel et al. (2003).	Upper Maastrichtian, Sanpetru Formation, Alba, Romania (Norman, 2004).	*NHMUK R3812, right metatarsal II.
Zalmoxes shqiperorum	Weishampel et al. (2003), Godefroit, Codrea & Weishampel (2009).	Upper Maastrichtian, Sanpetru Formation, Alba, Romania (Norman, 2004).	

Institutional abbreviations: BYU, Brigham Young University, Provo, Utah, United States; CM, Carnegie Museum of Natural History, Pittsburgh, Pennsylvania, United Sates; CMN, Canadian Museum of Nature, Ottawa, Ontario, Canada; IGM, Mongolian Institute of Geology, Ulaan Baatar, Mongolia; IRSNB, Royal Belgian Institute of Natural Sciences; JLUM, Geological Museum of the Jilin University, Changchun, Peoples Republic of China; MACN, Coleccion Paleontología de Vertebrados, Museo Argentino de Ciencias Naturales "Bernardino Rivadavia", Buenos Aires, Argentina; MB.R., Collection of Fossil Reptilia, Museum für Naturkunde (MfN), Berlin, Germany; MCF-PVPH, Museo Carmen Funes-Paleontología de Vertebrados, Plaza Huincul, Neuquén Province, Argentina; MCS, Museo Cinco Saltos; MOR, Museum of the Rockies, Bozeman, Montana, United States; MPM, Museo Padre Molina, Rio Gallegos, Santa Cruz, Argentina; MU,

Monash University, Melbourne, Victoria, Australia; MUCPv, Museo de Geologia y Paleontologia de la Universidad Nacional del Comahue, Paleontologia de Vertebrados, Neuquén Province, Argentina; MV, Museums Victoria, Melbourne, Victoria, Australia (formerly, National Museum of Victoria [NMV]); NHMUK, Natural History Museum, London, UK (formerly the British Museum of Natural History); OUMUK, Oxford University Museum of Natural History, Oxford, United Kingdom; QM, Queensland Museum, Brisbane, Queensland, Australia; ROM, Royal Ontario Museum, Toronto, Ontario, Canada; RSM, Royal Saskatchewan Museum, Regina, Canada; SAM, South African Museum, Cape Town, South Africa; SHN, Sociedade de História Natural, Torres Vedras, Portugal; UNPSJB, Universidad Nacional de la Patagonia 'San Juan Bosco', Argentina; USNM, National Museum of Natural History, Washington, D.C., United States; YPM, Yale Peabody Museum, New Haven, Connecticut, United States; ZDM, Zigong, Sichuan, Dinosaur Museum, Peoples Republic of China.

References

Bakker RT, Galton P, Siegwarth J, Filla J. 1990. A new latest Jurassic vertebrate fauna, from the highest levels of the Morrison Formation at Como Bluff, Wyoming. Part IV. The dinosaurs: a new *Othnielia*-like hypsilophodontid. *Hunteria* 2:8–19.

Barrett PM. 2016. A new specimen of *Valdosaurus canaliculatus* (Ornithopoda: Dryosauridae) from the Lower Cretaceous of the Isle of Wight, England *Memoirs of Museum Victoria* 74:29–48.

Barrett PM, Benson RBJ, Rich TH, Vickers-Rich P. 2011a. First spinosaurid dinosaur from Australia and the cosmopolitanism of Cretaceous dinosaur faunas. *Biology Letters* 7:933–936.

Barrett PM, Butler RJ, Twitchett RJ, Hutt S. 2011b. New material of *Valdosaurus canaliculatus* (Ornithischia: Ornithopoda) from the Lower Cretaceous of southern England. *Special Papers in Palaeontology* 86:131–163.

Bartholomai A, Molnar RE. 1981. *Muttaburrasaurus*, a new iguanodontid (Ornithischia: Ornithopoda) dinosaur from the Lower Cretaceous of Queensland. *Memoirs of the Queensland Museum* 20:319–349.

Brown CM, Boyd CA, Russell AP. 2011. A new basal ornithopod dinosaur (Frenchman Formation, Saskatchewan, Canada), and implications for late Maastrichtian ornithischian diversity in North America. *Zoological Journal of the Linnean Society* 163:1157–1198.

Brown CM, Evans DC, Ryan MJ, Russell AP. 2013. New data on the diversity and abundance of small-bodied ornithopods (Dinosauria, Ornithischia) from the Belly River Group (Campanian) of Alberta. *Journal of Vertebrate Paleontology* 33:495–520.

Butler RJ. 2005. The 'fabrosaurid' ornithischian dinosaurs of the Upper Elliot Formation (Lower Jurassic) of South Africa and Lesotho. *Zoological Journal of the Linnean Society* 145:175–218.

Butler RJ, Jin L, Jun C, Godefroit P. 2011. The postcranial osteology and phylogenetic position of the small ornithischian dinosaur *Changchunsaurus parvus* from the Quantou Formation (Cretaceous: Aptian–Cenomanian) of Jilin Province, north-eastern China. *Palaeontology* 54:667–683.

Calvo JO, Porfiri JD, Novas FE. 2007. Discovery of a new ornithopod dinosaur from the Portezuelo Formation (Upper Cretaceous), Neuquén, Patagonia, Argentina. *Arquivos do Museu Nacional, Rio de Janeiro* 65:471–483.

Cambiaso AV. 2007. Los ornitópodos e iguanodontes basales (Dinosauria, Ornithischia) del Cretácico de Argentina y Antártida Doctor of Philosophy Unpublished PhD thesis. Universidad de Buenos Aires.

Cooper MR. 1985. A revision of the ornithischian dinosaur *Kangnasaurus coetzeei* Haughton, with a classification of the Ornithischia. *Annals of the South African Museum* 95:281–317.

Coria RA, Calvo JO. 2002. A new iguanodontian ornithopod from Neuquén Basin, Patagonia, Argentina. *Journal of Vertebrate Paleontology* 22:503–509.

Coria RA, Salgado L. 1996. A basal iguanodontian (Ornithischia: Ornithopoda) from the Late Cretaceous of South America. *Journal of Vertebrate Paleontology* 16:445–457.

de Wit MCJ, Ward JD, Spaggiari R. 1992. A reappraisal of the Kangnas dinosaur site, Bushmanland, South Africa. *South African Journal of Science* 88:504–507.

Eberth DA, Kobayashi Y, Lee Y-N, Mateus O, Therrien F, Zelenitsky DK, Norell MA. 2009. Assignment of *Yamaceratops dorngobiensis* and associated redbeds at Shine Us Khudag (eastern Gobi, Dorngobi Province, Mongolia) to the redescribed Javkhlant Formation (Upper Cretaceous). *Journal of Vertebrate Paleontology* 29:295–302.

Escaso F, Ortega F, Dantas P, Malafaia E, Silva B, Gasulla JM, Mocho P, Narváez I, Sanz JL. 2014. A new dryosaurid ornithopod (Dinosauria, Ornithischia) from the Late Jurassic of Portugal. *Journal of Vertebrate Paleontology* 34:1102–1112.

Forster CA. 1990. The postcranial skeleton of the ornithopod dinosaur *Tenontosaurus tilletti*. *Journal of Vertebrate Paleontology* 10:273–294.

Galton PM. 1974. The ornithischian dinosaur *Hypsilophodon* from the Wealden of the Isle of Wight. *Bulletin of the British Museum (Natural History) Geology* 25:1–152.

Galton PM. 1981. *Dryosaurus*, a hypsilophodontid dinosaur from the Upper Jurassic of North America and Africa. Postcranial skeleton. *Paläontologische Zeitschrift* 55:271–312.

Galton PM. 2014. Notes on the postcranial anatomy of the heterodontosaurid dinosaur *Heterodontosaurus tucki*, a basal ornithischian from the Lower Jurassic of South Africa. *Revue de Paleobiologie* 33:97–141.

Galton PM, Jensen JA. 1973. Skeleton of a hypsilophodontid Dinosaur (*Nanosaurus* (?) *rex*) from the Upper Jurassic of Utah. *Brigham Young University, Geology Studies* 20:137–157.

Galton PM, Powell HP. 1980. The ornithischian dinosaur *Camptosaurus prestwichii* from the Upper Jurassic of England. *Palaeontology* 23:411–433.

Galton PM, Taquet P. 1982. *Valdosaurus*, a hypsilophodontid dinosaur from the Lower Cretaceous of Europe and Africa. *Geobios* 15:147–159 + 141 plate.

Gilmore CW. 1909. Osteology of the Jurassic reptile *Camptosaurus*, with a revision of the species of the genus, and descriptions of two new species. *Proceedings of the United States National Museum* 36:197–332.

Gilmore CW. 1915. Osteology of *Thescelosaurus*, an orthopodous dinosaur from the Lance Formation of Wyoming. *Proceedings of the United States National Museum* 49:591–616.

Godefroit P, Codrea VA, Weishampel DB. 2009. Osteology of *Zalmoxes shqiperorum* (Dinosauria, Ornithopoda), based on new specimens from the Upper Cretaceous of Nălaţ-Vad (Romania). *Geodiversitas* 31:525–553.

Gross JD, Rich TH, Vickers-Rich P. 1993. Dinosaur bone infection: chronic osteomyelitis in a hypsilophodontid dinosaur in Early Cretaceous, polar Australia. *National Geographic Research and Exploration* 9:286–293.

Han F-L, Barrett PM, Butler RJ, Xu X. 2012. Postcranial anatomy of *Jeholosaurus shangyuanensis* (Dinosauria, Ornithischia) from the Lower Cretaceous Yixian Formation of China. *Journal of Vertebrate Paleontology* 32:1370–1395.

Haughton SH. 1915. On some dinosaur remains from Bushmanland. *Transactions of the Royal Society of South Africa* 5:259–264.

He X, Cai K. 1984. *The Middle Jurassic dinosaurian fauna from Dashanpu, Zigong, Sichuan, Volume 1*. Chengdu, Sichuan: Sichuan Scientific and Technological Publishing House.

Herne MC. 2009. Postcranial osteology of *Leaellynasaura amicagraphica* (Dinosauria; Ornithischia) from the Early Cretaceous of southeastern Australia. *Sixty-ninth Annual Meeting Society Of Vertebrate Paleontology* 29:77A.

Herne MC. 2014. Anatomy, Systematics and Phylogenetic Relationships of the Early Cretaceous Ornithopod Dinosaurs of the Australian-Antarctic Rift System PhD dissertation. The University of Queensland.

Herne MC, Tait AM, Salisbury SW. 2016. Sedimentological reappraisal of the *Leaellynasaura amicagraphica* (Dinosauria, Ornithopoda) holotype locality in the Lower Cretaceous of Victoria, Australia with taphonomic implications for the taxon. *New Mexico Museum of Natural History and Science Bulletin* 71:121–148.

Hübner TR. 2011. Ontogeny in *Dysalotosaurus lettowvorbecki* Doctor of Philosophy Dissertation. Ludwig-Maximilians-Universität.

Hulke JW. 1882. An attempt at a complete osteology of *Hypsilophodon foxii*: a British Wealden dinosaur. *Philosophical Transactions of the Royal Society of London* 172:1035–1062.

Ibiricu LM, Martínez RD, Luna M, Casal GA. 2014. A reappraisal of *Notohypshilophodon comodorensis* (Ornithischia: Ornithopoda) from the Late Cretaceous of Patagonia, Argentina. *Zootaxa* 3786:401–422.

Janensch W. 1955. Der ornithopode *Dysaltosaurus* der Tendaguruschichten. *Palaeontographica (Supplement 7)* 3:105–176.

Krumenacker LJ. 2017. Osteology, phylogeny, taphonomy, and ontogenetic histology of *Oryctodromeus cubicularis*, from the Middle Cretaceous (Albian-Cenomanian) of Montana and Idaho. Unpublished PhD Dissertation. Montana State University.

Makovicky PJ, Kilbourne BM, Sadleir RW, Norell MA. 2011. A new basal ornithopod (Dinosauria, Ornithischia) from the Late Cretaceous of Mongolia. *Journal of Vertebrate Paleontology* 31:626–640.

Martinez RD. 1998. *Notohypsilophodon comodorensis*, gen. et sp. nov., un Hypsilophodontidae (Ornithischia: Ornithopoda) del Cretacico Superior de Chubut, Patagonia central, Argentina. *Acta Geologica Leopoldensia XXI* 46-47:119–135.

McDonald AT. 2011. The taxonomy of species assigned to *Camptosaurus* (Dinosauria: Ornithopoda). *Zootaxa* 2783:52–68.

Molnar RE. 1996. Observations on the Australian ornithopod dinosaur *Muttaburrasaurus*. *Memoirs of the Queensland Museum* 39:639–652.

Norman DB. 1980. On the ornithischian dinosaur *Iguanodon bernissartensis* from the Lower Cretaceous of Bernissart (Belgium). *Instiut Royal des Sciences Naturelles de Belgique, Memoire* 178:1–105.

Norman DB. 1986. On the anatomy of *Iguanodon atherfieldensis* (Ornithischia: Ornithopoda). *Bulletin de l'Institut Royal des Sciences Naturelles de Belgique: Sciences de la Terre* 56:281–372.

Norman DB. 2004. Basal Iguanodontia. In: Weishampel DB, Dodson P, Osmólksa H, eds. *The Dinosauria*. 2 ed. Berkley, London: University of California Press, 413–437.

Norman DB, Sues H-D, Witmer LM, Coria RA. 2004. Basal Ornithopoda. In: Weishampel DB, Dodson P, Osmólksa H, eds. *The Dinosauria (2nd ed)*. Berkley, London: University of California Press, 393–412.

Novas FE. 2009. *The Age of Dinosaurs in South America*. Bloomington: Indiana University Press.

Novas FE, Cambiaso AV, Ambrosio A. 2004. A new basal iguanodontian (Dinosauria, Ornithischia) from the Upper Cretaceous of Patagonia. *Ameghiniana* 41:75–82.

Ostrom JH. 1970. Stratigraphy and paleontology of the Cloverly Formation (Lower Cretaceous) of the Bighorn Basin Area, Wyoming and Montana. *Bulletin of the Peabody Museum of Natural History* 35:1–234.

Parks WA. 1926. *Thescelosaurus warreni*, a new species of orthopodous dinosaur from the Edmonton Formation of Alberta. *University of Toronto Studies, Geological Series* 21:1–42.

Peng G. 1992. Jurassic ornithopod *Agilisaurus louderbacki* (Ornithopoda: Fabrosauridae) from Zigong, Sichuan, China. *Vertebrata PalAsiatica* 30:39–53.

Rich TH, Rich PV. 1989. Polar dinosaurs and biotas of the Early Cretaceous of southeastern Australia. *National Geographic Society Research Reports* 5:15–53.

Rich TH, Vickers-Rich P. 1999. The Hypsilophodontidae from southeastern Australia. In: Tomida Y, Rich TH, Vickers-Rich P, eds. *Proceedings of the Second Gondwanan Dinosaur Symposium*. Tokyo: National Science Museum, 167–180.

Rozadilla S, Agnolin FL, Novas FE, Aranciaga Rolando AM, Motta MJ, Lirio JM, Isasi MP. 2016. A new ornithopod (Dinosauria, Ornithischia) from the Upper Cretaceous of Antarctica and its palaeobiogeographical implications. *Cretaceous Research* 57:311–324.

Salgado L, Coria RA, Heredia SE. 1997. New materials of *Gasparinisaura cincosaltensis* (Ornithischia, Ornithopoda) from the Upper Cretaceous of Argentina. *Journal of Paleontology* 71:933–940.

Santa Luca AP. 1980. The postcranial skeleton of *Heterodontosaurus tucki* (Reptilia, Ornithischia) from the Stormberg of South Africa. *Annals of the South African Museum* 79:159–211.

Scheetz RD. 1999. Osteology of *Orodromeus makelai* and the phylogeny of basal ornithopod dinosaurs Doctor of Philosophy Unpublished PhD dissertation. Montana State University.

Sereno PC. 1991. *Lesothosaurus*, "Fabrosaurids," and the early evolution of Ornithischia. *Journal of Vertebrate Paleontology* 11:168–197.

Sereno PC. 2012. Taxonomy, morphology, masticatory function and phylogeny of heterodontosaurid dinosaurs. *ZooKeys* 226:1–225.

Sternberg CM. 1940. *Thescelosaurus edmontonensis*, n. sp., and classification of the hypsilophodontidae. *Journal of Paleontology* 14:481–494.

Taquet P. 1976. *Geologie et paleontologie du gisement de Gadoufaoua (Aptien du Niger)*. Paris: Editions du Centre National de la Recherche Scientifique.

Thulborn RA. 1972. The post cranial skeleton of the Triassic ornithischian dinosaur *Fabrosaurus australis*. *Palaeontology* 15:29–60.

Varricchio DJ, Martin AJ, Katsura Y. 2007. First trace and body fossil evidence of a burrowing, denning dinosaur. *Proceedings of the Royal Society of London B* 274:1361–1368.

Weishampel DW, Jianu C-M, Csiki Z, B. ND. 2003. Osteology and phylogeny of *Zalmoxes* (n. g.), an unusual Euornithopod dinosaur from the latest Cretaceous of Romania. *Journal of Systematic Palaeontology* 1:65–123.
