# Supplementary material for: A new small-bodied ornithopod (Dinosauria, Ornithischia) from a deep, high-energy Early Cretaceous river of the Australian–Antarctic rift system
Source: PeerJ. 2018 Jan 11;5:e4113. doi: 10.7717/peerj.4113 (PMC5767335; doi:10.7717/peerj.4113)
Supplement: Supplemental Information 11 — Dorsoventral heights: ‘a’ measured vertically from dorsal tip of spinal process to centre of transverse process; ‘b,’ measured vertically from dorsal tip of spinal process to ventral-most margin of centrum; and ‘c,’ measured from dorsal tip of spinal process to ventral tip of haemal process. For taxa where height information is not shown, proportions were estimated from figures within the literature (see sources, Table 1). Abbreviations: Ca, caudal vertebral position; ?, unknown or estimated caudal position. [file peerj-06-4113-s011.pdf]

Table S2. Comparative dorsoventral proportions of the anterior caudal vertebrae for selected ornithopods (graphical presentation, Fig. 28).

		Height mm			Proportion %	
**Taxon**	**Ca**	**a**	**b**	**c**	**a/b**	**a/c**
Diluvicursor pickeringi, holotype, NMV P221080	3?	8.3	20.0	50.0 e	42	17
NMV P228342	1-2?	22.5	47.5		47	-
Parksosaurus warreni (ROM 804)	4	36.0	65.0	116.5	55	31
Dysalotosaurus lettowvorbecki (BM.R.1587.4)	4				56	-
Valdosaurus canaliculatus (IWCMS 2013.175)	3				56	-
Jeholosaurus shangyuanensis (IVPP V15939)	3				59	-
Eousdryosaurus nanohallucis (SHN(JJS)-170)	3				60	36
Jeholosaurus shangyuanensis (IVPP V12542)	4				60	32
Haya griva (IGM 100/2015)	3				60	34
Orodromeus makelai (MOR 623)	?				61	-
Thescelosaurus neglectus (USNM 7757)	5				62	37
Hypsilophodon foxii (NHM R196)	4				70	37
Mantellisaurus atherfieldensis (IRSNB 1551)	4				70	46
Camptosaurus dispar (USNM 4282)	2				70	-
Tenontosaurus tilletti (AMNH 3040)	3				73	42

Notes: Dorsoventral heights: 'a', dorsal tip of spinal process to centre of transverse process; 'b', dorsal tip of spinal process to ventral-most margin of centrum; and 'c', dorsal tip of spinal process to ventral tip of haemal process. For specimen and literature sources, see Table S1. For taxa where heights are not shown, proportions were estimated from figures within the literature sources (Table S1). For taxa where proportion 'a/c' is not shown (-), haemal arch height ('c') is unknown. Abbreviations: Ca, caudal vertebral position; ?, estimated caudal position.
