# Supplementary material for: A new small-bodied ornithopod (Dinosauria, Ornithischia) from a deep, high-energy Early Cretaceous river of the Australian–Antarctic rift system
Source: PeerJ. 2018 Jan 11;5:e4113. doi: 10.7717/peerj.4113 (PMC5767335; doi:10.7717/peerj.4113)
Supplement: Supplemental Information 12 — Notes: the first pedal phalanges (pd I-1) in D. lettowvorbecki and Eousdryosaurus nanohallucis are alternative identifications using the dimensions for the bones identified as first metatarsals (following Galton, 1981; Escaso et al., 2014). Measurements of elements from literature sources in Table S1. Abbreviations: DDH, distal dorsoplantar height; DTW, distal transverse width; (e), estimated from articulating surface of adjoining bone; l, left; mt #, metatarsal position; pd #, phalanx and position; PDL, proximodistal length; PDH, proximal dorsoplantar height; PTW, proximal transverse width; r, right. [file peerj-06-4113-s012.pdf]

**Table S3.** Pedal digit proportions for selected ornithischians (graphical presentation, Fig. S6).

| Taxon | PDL pd I-1/mt III | DDH mt I/DDH mt II | DTW mt I/mt II | PTW pd I-1/pd II-1 | PDH pd I-1/pd II-1 | PDH pd I-2/DDH mt II |
|---|---|---|---|---|---|---|
| ***Anabisetia saldiviai* (PVPH-75) l** | 22% | 34% | 42% | 57% | 36% | 29% |
| ***Agilisaurus louderbacki* (ZDM 6011)** | 29% | 87% | 89% | 66% | 85% | 80% |
| ***Camptosaurus dispar* (USNM 4277)** | 20% | | 41% | | | |
| ***Changchunsaurus parvus* (JLUM L0403-j-Zn2)** | 28% | 91% | 71% | | | |
| ***Diluvicursor pickeringi*, holotype, NMV P221080 r** | 2 7 % | 50% | 62% | 56% | 50% | 48% |
| ***Heterodontosaurus tucki* (SAM-PK-K1332)** | 25% | | 76% | 73% | | |
| ***Hypsilophodon foxii* (NHM R196) r** | 35% | 75% | 87% | 100% | 90% | |
| ***Jeholosaurus shangyuanensis* (IVPP V15939) l** | 21% | 68% | 77% | 85% | 77% | 72% |
| **NMV P185992 r** | | | 63% | 77% | | |
| **NMV P186047 l** | 19% | | 50% l | 46% (e) | | |
| ***Orodromeus makelai* (MOR 530)** | 25% | | 80% | 83% | | |
| ***Othnielosaurus consors* (ROM 46240)** | 30% | | 92% | 83% | 91% | 81% |
| ***Parksosaurus warreni* (ROM 804)** | 33% r | 86% l | 99% r | 90% r | 78% l | 70% l |
| ***Thescelosaurus assiniboiensis* (RSM P 1225.1) l** | 53% | | 67% | 73% | | |

**Notes:** Specimen and data sources, Table S1. **Abbreviations. Anatomical:** (e), estimated from articulating surface of adjoining bone; l, left; mt #, metatarsal number; pd #, phalanx position; r, right. **Distance:** DDH, distal dorsoplantar height; DTW, distal transverse width; PDL, proximodistal length; PDH, proximal dorsoplantar height; PTW, proximal transverse width.
