# Supplementary material for: A new small-bodied ornithopod (Dinosauria, Ornithischia) from a deep, high-energy Early Cretaceous river of the Australian–Antarctic rift system
Source: PeerJ. 2018 Jan 11;5:e4113. doi: 10.7717/peerj.4113 (PMC5767335; doi:10.7717/peerj.4113)
Supplement: Supplemental Information 13 [file peerj-06-4113-s013.docx]

**TABLE S4:** Character states of new OTUs added to the matrices published by Boyd (2015), Dieudonné et al. (2016), and Han et al. (2017, in press).

Boyd, 2015 (unadjusted dataset): *Diluvicursor pickeringi*

??????????????????????????????????????????????????????????????????????????????????????????????????????????????????????????????????????????????????????01[0 1]???????????????????????????????????????????????????????????????????????????2???130211101?0011?????????

Boyd, 2015 (dataset corrected for Victorian OTUs): *Diluvicursor pickeringi*

??????????????????????????????????????????????????????????????????????????????????????????????????????????????????????????????????????????????????????01[0 1]???????????????????????????????????????????????????????????????????????????2???130211101?0011?????????

Boyd, 2015 (dataset corrected for Victorian OTUs): NMV P186047 (formerly as postcranial scores of *Leaellynasaura amicagraphica*)

?????????????????????????????????????????????????????????????????????????????????????????????????????????????????????????????????????????????????????????????????????????????????????????????????2003?1000100?0000?0?101202?0??000??210??00010????0001000??????

Dieudonné et al. 2016: *Diluvicursor pickeringi*

??????????????????????????????????????????????????????????????????????????????????????????????????????????????????????????????????????????????????????????????????????????????10???1????0??????????????????????????????????????????????????????????????????????????????1?1100011111?01?1101??????

Han et al. 2017, in press: *Diluvicursor pickeringi*

???????????????????????????????????????????????????????????????????????????????????????????????????????????????????????????????????????????????????????????????????????????????????????????????????????????????????????????????????????????????????????10?00000??0-??????????????????????????????????????????????????????????????????????????????????????????????????????2?010211001?11000??1

**References**:

Boyd CA. 2015. The systematic relationships and biogeographic history of ornithischian dinosaurs. PeerJ 3:e1523. DOI 10.7717/peerj.1523

Dieudonné P-E, Tortosa T, Torcida Fernández-Baldor F, Canudo JI, and Díaz-Martínez I. 2016. An unexpected early rhabdodontid from Europe (Lower Cretaceous of Salas de los Infantes, Burgos Province, Spain) and a re-examination of basal iguanodontian relationships. PLoS ONE 11:e0156251. doi:10.1371/journal.pone.0156251

Han F, Forster CA, Xu X, and Clark JM. 2017, in press. Postcranial anatomy of *Yinlong downsi* (Dinosauria: Ceratopsia) from the Upper Jurassic Shishugou Formation of China and the phylogeny of basal ornithischians. *Journal of Systematic Palaeontology* Online early view, 29 pp.
